# Characterization of Different Types of Micro-Fission and Micro-Ionization Chambers Under X-Ray Beams

**DOI:** 10.3390/s25061862

**Published:** 2025-03-17

**Authors:** Juan Antonio Moreno-Pérez, Álvaro Marchena, Pablo Araya, Jesús J. López-Peñalver, Juan Alejandro de la Torre, Antonio M. Lallena, Santiago Becerril, Marta Anguiano, Alberto J. Palma, Miguel A. Carvajal

**Affiliations:** 1 Electronic and Chemical Sensing Solutions (ECsens), CITIC-UGR, Department of Electronics and Computer Technology, University of Granada, E-18014 Granada, Spain; juanantoniomp@ugr.es (J.A.M.-P.); carvajal@ugr.es (M.A.C.); 2 IFMIF-DONES España, Gran Vía de Colón, 48, E-18010 Granada, Spain; alvaro.marchena@ifmif-dones.es (Á.M.); pablo.araya@ifmif-dones.es (P.A.); santiago.becerril@ifmif-dones.es (S.B.); 3 Experimental Radiology Research Unit, Scientific Instrumentation Center, University of Granada, E-18071 Granada, Spain; jjpenalver@ugr.es; 4 Department of Atomic, Molecular and Nuclear Physics, University of Granada, E-18071 Granada, Spain; juanalejandro.delatorre.gonzalez@gmail.com (J.A.d.l.T.); lallena@ugr.es (A.M.L.); mangui@ugr.es (M.A.); 5 Instituto de Investigación Biosanitaria (ibs.GRANADA), Complejo Hospitalario Universitario de Granada/University of Granada, E-18016 Granada, Spain; 6 Sport and Health University Research Institute (iMUDS), University of Granada, E-18071 Granada, Spain

**Keywords:** micro-ionization chamber, micro-fission chamber, reproducibility, fusion reactors

## Abstract

Various models of ionization and fission chambers for ionizing radiation detection, designed to operate under harsh conditions such as those found in fusion reactors or particle accelerators, have been experimentally characterized and numerically simulated. These models were calibrated using a photon beam in the X-ray spectrum. Irradiations were performed at the Biomedical Research Center of the University of Granada (CIBM) with a bipolar metal-ceramic X-ray tube operating at a voltage of 150 kV and a dose rate ranging from 0.05 to 2.28 Gy/min. All detectors under study featured identical external structures but varied in detection volume, anode configuration, and filling gas composition. To assess inter- and intra-model response variations, the tested models included 12 micro-ionization chambers (CRGR10/C5B/UG2), 3 micro-fission chambers (CFUR43/C5B-U5/UG2), 8 micro-fission chambers (CFUR43/C5B-U8/UG2), and 3 micro-fission chambers (CFUR44/C5B-U8/UG2), all manufactured by Photonis (Merignac, France). The experimental setup was considered suitable for the tests, as the leakage current was below 20 pA. The optimal operating voltage range was determined to be 130–150 V, and the photon sensitivities for the chambers were measured as 29.8 ± 0.3 pA/(Gy/h), 43.0 ± 0.8 pA/(Gy/h), 39.2 ± 0.3 pA/(Gy/h), and 96.0 ± 0.9 pA/(Gy/h), respectively. Monte Carlo numerical simulations revealed that the U layer in the fission chambers was primarily responsible for their higher sensitivities due to photoelectric photon absorption. Additionally, the simulations explained the observed differences in sensitivity based on the filling gas pressure. The detectors demonstrated linear responses to dose rates and high reproducibility, making them reliable tools for accurate determination of ionizing photon beams across a range of applications.

## 1. Introduction

Beam diagnostics has always been fundamental in radiation environments [1,2,3] such as nuclear reactors, particle accelerators, and similar facilities, especially under extreme conditions as well as biomedical applications. It helps to ensure the success of experimental outcomes, the safety of personnel, and the integrity of the equipment [4]. For this type of monitoring, a wide variety of detectors are commonly employed, such as gamma thermometers [5], self-powered neutron detectors (SPNDs) [6], ionization chambers, and fission chambers [7], among others [8].

Ionization chambers (ICs) are particularly sensitive to gamma radiation and are widely used for measuring high radiation levels in various nuclear applications [9]. They operate by collecting ion pairs created by radiation [10]. Fission chambers (FCs) are sensitive to both gamma and neutron radiation, detecting the fission fragments produced when neutrons interact with a fissile material within the chamber. Depending on this material, they can be more sensitive to thermal neutrons (e.g., U-235) [11] or fast neutrons (e.g., U-238) [12]. Both detectors can be designed to operate with either continuous or pulsed radiation beams [13,14] by adjusting fabrication parameters such as the internal gas pressure, thereby varying the sensitivity and response characteristics [15]. Additionally, they are influenced by the polarization voltage applied across their electrodes [16,17,18]. This biasing determines the charges from ionization collected at the electrodes. Detectors are typically operated in the plateau region of their response curve, where changes in applied voltage minimally affect the response to dose rate.

Pairing ionization and fission chambers can effectively discriminate the neutron contribution in a mixed field with a gamma component [19]. When both detectors share the same geometry and composition, except for the fissile material in the anode of the fission chamber, placing them together allows for accurate discrimination between neutron and gamma radiation [20]. The ionization chamber provides a baseline measurement of the gamma component. The fission chamber measures the total radiation from the gamma and neutron components, as it shares the same method for detecting gammas, and additionally detects neutrons through the fission process induced in the fissile material. The neutron contribution can be extracted from these combined readings. This is highly relevant for installations such as particle accelerators for neutron diagnostics [21].

This study presents a comparative analysis with an experimental characterization and numerical simulation of various models of ionization and fission micro-chambers under photon beams of lower energy and lower dose rate than their usual applications. Each model shares the external configuration but differs in the detection volume, the filling gas composition, pressure, and fissile material in the case of the fission chambers. The aim is to assess their performance and reproducibility, focusing on the sensitivity to photons emitted by an X-ray source [22]. This represents a highly controlled irradiation setup, preventing the study complexity when mixed fields are applied. In addition, Monte Carlo simulations of the devices have been carried out for explaining the differences in response to radiation between both types of microchambers. This evaluation provides valuable insights into the suitability of the detectors for diverse operating conditions, thus laying the groundwork for their practical deployment. In the present work, the focus is on the differences in response, both intra- and inter-models, under the same conditions, performing a more comprehensive statistical analysis based on a significantly larger sample size.

## 2. Materials and Methods

### 2.1. Samples and Experimental Setup

Four different types of detectors were studied, comprising 12 micro-ionization chambers of one model and 14 micro-fission chambers of three different models (Photonis, Mérignac, France) (see Table 1). As mentioned, these detectors share the same external structure, with variations in the composition and pressure of the filling gas, and the fissile material in the anode of the fission chambers. Active volume has a thickness of 0.25 mm. The central anode has a radius of 1 mm, and it is made of steel. In the case of the fission chambers, it is coated with an 8 μm layer of U (300 µg). The radius of the overall envelope is 1.5 mm and is made of steel. Moreover, an ionization chamber has a detection volume, V_d_, with a length, L_d_, of 14 mm, whereas for the fission chambers, this dimension is shorter, 10 mm. Therefore, the detection volume is different in both types of chambers. All models use argon at a pressure of 5 bar as their filling gas, except for the model CFUR44/C5B-U8/UG2, which contains a mixture of argon with 4% nitrogen at a higher pressure of 15 bar, thus increasing its sensitivity.

Irradiations were conducted at the Biomedical Research Center of the University of Granada (CIBM, Granada, Spain), with a bipolar metal–ceramic X-ray source from Comet Yxlon (Hamburg, Germany), capable of emitting photons within the X-ray energy spectrum, with adjustable voltage and current up to 320 kV and 22.5 mA, respectively. The voltage was set at 150 kV for all tests, and the current was varied to modulate the dose rate. A PTW 23342 ionization chamber (PTW, Freiburg, Germany) was the reference detector for dose rate determination. This IC has shown excellent performance under X-rays at a voltage source higher than 100 kV [23,24].

**Figure 1:**
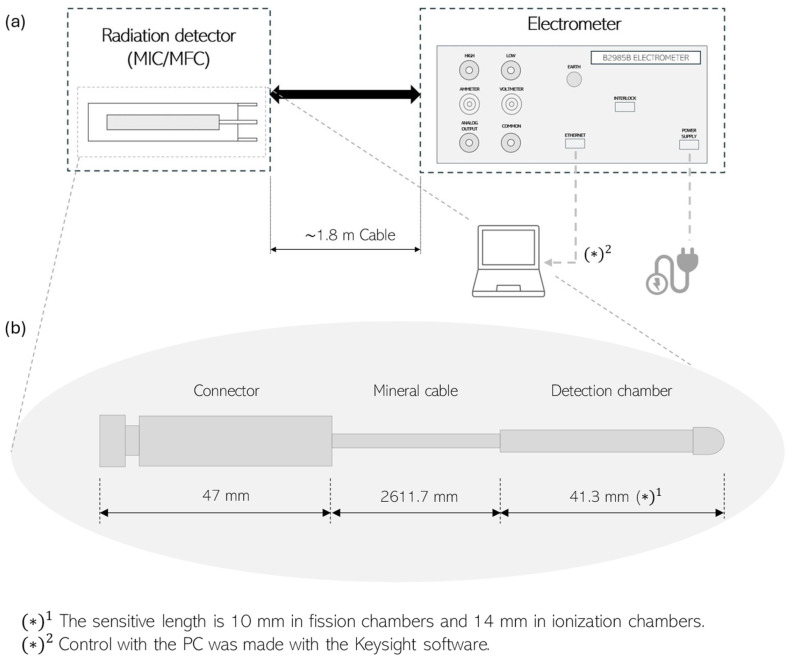
Setup for measurements with the chambers (Photonis, Mérignac, France): (**a**) complete setup and (**b**) dimensions of the detectors given in mm.

A Keithley B2985B electrometer (Tektronix, Cleveland, OH, USA) was configured for current acquisition from the detectors. This electrometer offers a minimum current resolution of 0.01 fA and can bias devices up to ±1000 V. To minimize electromagnetic interferences, the detectors and the electrometer were connected through balanced triaxial transmission lines (Figure 1a). The external structure of the detectors is nearly identical (Figure 1b), differing only in the sensitive part of the chamber.

**Figure 2:**
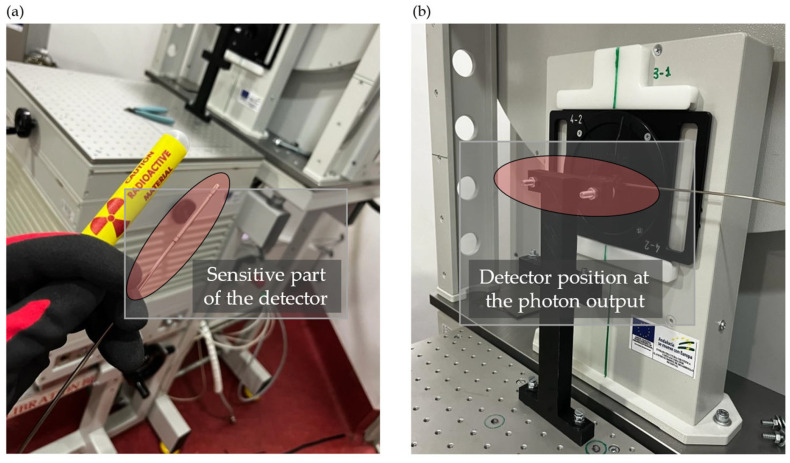
Laboratory of the CIBM for X-ray irradiation: (**a**) sensitive part of the detector in the foreground. X-ray tube with the support structure anchored to the bench in the background. (**b**) Detector placed in the irradiation position at the output of the source.

Two connection interfaces were developed. One serves as an adapter from the coaxial SMA of the chambers to the triaxial line, and the other acts as an interface for the electrometer connection with the chamber. Both interfaces were designed to be housed within metal enclosures to maintain signal shielding. Additionally, a commercial BNC-to-banana adapter was used in the connection. The electrometer was located in the control room, close to the irradiation room where the detectors were positioned and establishing the connection through a cable conduit. The total length of the cable used for the connection was approximately 4.5 m. The electrometer was controlled remotely via Ethernet. Devices under tests (DUTs) were positioned on a specially designed 3D-printed support, affixed to an adjustable bench through screws. The sensitive part of the detectors was placed at 224.8 ± 0.1 mm from the photon output and secured with ties to prevent excessive mechanical stress and facilitate device replacement (Figure 2). No build-up materials were used in front or behind the detectors, directly facing the X-ray beam. This setup is usual in this type of detectors in typical applications under harsh radiation environment [20,21].

The campaign was divided into three different tests:

1. Measurement of the leakage current of some detectors to verify the setup proposed.
2. Study of the IV curve under stable radiation conditions to determine the plateau region.
3. Characterization of the detectors to a photon beam in the X-rays spectrum.

The humidity and temperature conditions were monitored during the two-day campaign, with maximum variations recorded of ±1% and ±0.7 °C, respectively. These conditions have been considered as constant during the experiment; therefore, no corrections have been applied to the detectors.

The experimental uncertainties were analyzed according to the JCGM 100 report [25], using a coverage factor of k = 1. Type A uncertainties arise from the statistical variability of the data for each IC or FC unit. Given that this study focuses on the reproducibility of the four chambers, each sensitivity value was determined from the slope of the least-squares fit of the measured induced current as a function of the dose rate, with its corresponding uncertainty. The sensitivity and uncertainty for each chamber model were obtained by averaging the individual sample values, as recommended in [25]. Type B uncertainty in this experiment stems from the electrometer, with a value of 0.2% in the current range used, as specified by the manufacturer. The various uncertainties of the readouts were combined in quadrature.

### 2.2. Monte Carlo Simulations

**Figure 3:**
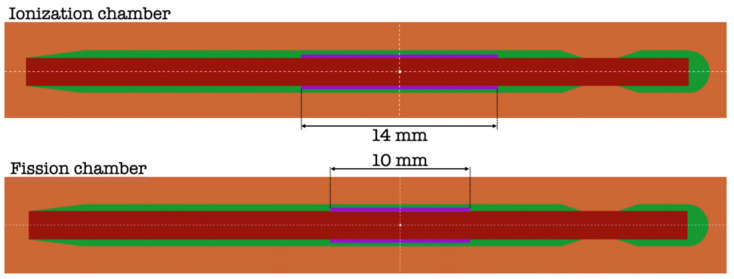
Scheme of the chambers used in the Monte Carlo simulations carried out. Central anode is represented in dark red and steel envelope in green.

To a better understanding of the experimental results, a series of Monte Carlo simulations have been performed using the code PENELOPE [26]. The geometries of the chambers are outlined in Figure 3. They have been built up according to the technical information available (Photonis, Mérignac, France). Both have a very similar shape, the only differences occurring in the active volume (in violet in the figure), which is filled with Ar (or Ar and 4% N_2_). In the case of the ionization chamber, it has a length of 14 mm, while in the case of the fission chamber, it has 10 mm. In both cases, the active volume has a thickness of 0.25 mm. The central anode (in dark red in Figure 3) has a radius of 1 mm, and it is made of steel. In the case of the fission chamber, it is coated with 8 μm layer of U. The radius of the overall envelope is 1.5 mm and is made of steel (in green in Figure 3). Actual gas compositions and pressures have been included in the filling gas for the simulations. Additional simulations have been completed by simplifying the geometry of the chambers, leaving just the region of the active volume. In this way, the possible effects due to the elements external to the area of the active volume of the chambers have been eliminated.

In the simulations, chambers have been irradiated under conditions similar to those of the experimental measurements by situating the chamber within an air phantom at a depth of 22.5 cm. A plane-parallel beam with a size of 6 cm × 6 cm and an X-ray spectrum corresponding to 150 kVp was considered. This X-ray spectrum was generated using the Python package SpekPy v2.0, trying to reproduce the experimental source used during the irradiation [27,28]. Also, a ^60^Co beam of the same geometrical characteristics has been also included in the simulations for comparison purposes. In this case, photons with 1.25 MeV (the average photon energy of this radioisotope) were emitted from the source. This is a common simplification when considering ^60^Co sources in Monte Carlo simulations, in general, and specifically for studying the response of ionization chambers [29].

In PENELOPE, photons are simulated in a detailed way, that is, interaction by interaction in chronological order. Electrons and positrons are simulated by using a mixed class-II procedure in which interactions are classified as “hard” or “soft”, depending on the energy loss and/or change of direction suffered by the interacting particle. The simulation parameters employed for all the materials in the simulation geometry were C_1_ = C_2_ = 10^−3^, W_CC_ = 10 keV, and W_CR_ = 1 keV. The meaning of these parameters can be found in the PENELOPE manual [22]: C_1_ sets the average angular deflection occurred between two consecutive hard collisions; C_2_ determines the maximum fractional energy loss that is permitted between two hard collisions, and W_cc_ and W_cr_ indicate the threshold energies for hard inelastic interactions and hard bremsstrahlung emission, respectively. Additionally, particle transport is controlled by the absorption energies: E_e−_, E_e+_, and E_gamma_, representing the energies at which the simulation of electrons, positrons, and photons, respectively, is discontinued, with the particles being absorbed in the material through which they were moving. In our simulations, these absorption energies were chosen to be 10 keV for electrons and positrons and 1 keV for photons, in all materials. Finally, and recommended in the code manual, D_SMAX_ was fixed to one-tenth of the shorter dimension of each element in the geometry. This parameter defines the maximum size of the particle track steps and plays a crucial role in geometries that include thin structures, as is the case. The values adopted in our simulations are within the ranges recommended in the PENELOPE manual.

## 3. Results

### 3.1. Experimental Results

#### 3.1.1. Leakage Current

**Figure 4:**
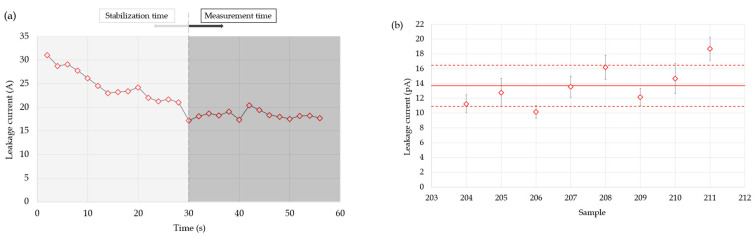
Leakage current analysis: (**a**) leakage current transient of the micro-ionization chamber CRGR10/C5B/UG2-211 and (**b**) average leakage current of the micro-ionization chambers CRGR10/C5B/UG2 from 204 to 211. Average is represented by a solid line and the ±1 SD range indicated by dashed lines.

The leakage current was measured to evaluate the background response of the system. These tests were performed without radiation, with the DUTs biased at 150 V. The electrometer was configured in the 200 pA range to increase the resolution and avoid errors due to scale changes. The signal was sampled at a frequency of 0.5 Hz, and the integration time was set as stable, automatically adjusted to a longer aperture time to reduce the noise. The tests were performed with the ionization chambers 204 to 211. The leakage current was determined as the average of the measurements taken after a 30 s stabilization period (Figure 4a).

The leakage current obtained with each sample is shown in Figure 4b, with the average represented by a solid line and the ±1 SD range indicated by dashed lines. The average was 14.2 ± 0.9 pA, with a maximum value of 18.7 ± 1.3 pA, so the setup can be considered suitable for measuring the induced current with high accuracy and low noise level arising from the connections. This is consistent with the data provided by the manufacturer, which guarantees a leakage current of less than 150 pA for their detectors.

#### 3.1.2. IV Curve

The IV curve of the devices was analyzed to identify and define the plateau region, thereby determining the optimal voltage range for their operation. Two voltage sweeps were performed, as shown in Table 2. The conditions of the X-ray tube were set at 150 kV and 9 mA.

**Figure 5:**
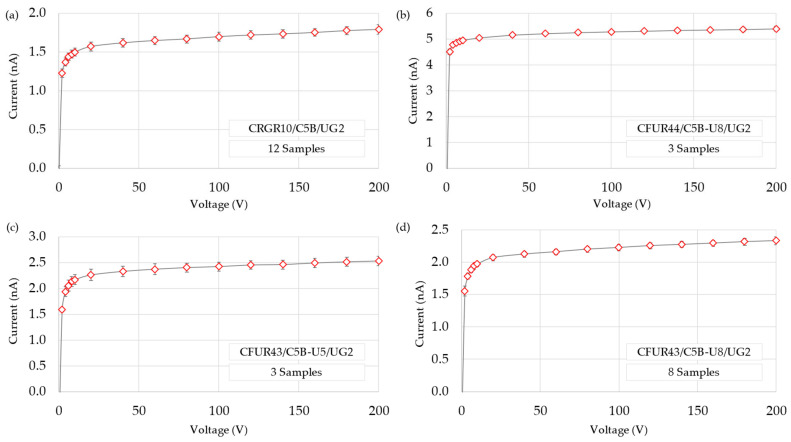
IV curves obtained with all the DUTs: (**a**) micro-ionization chamber CRGR10/C5B/UG2, (**b**) micro-fission chamber CFUR44/C5B-U8/UG2, (**c**) micro-fission chamber CFUR43/C5B-U5/UG2, and (**d**) micro-fission chamber CFUR43/C5B-U8/UG2.

**Figure 6:**
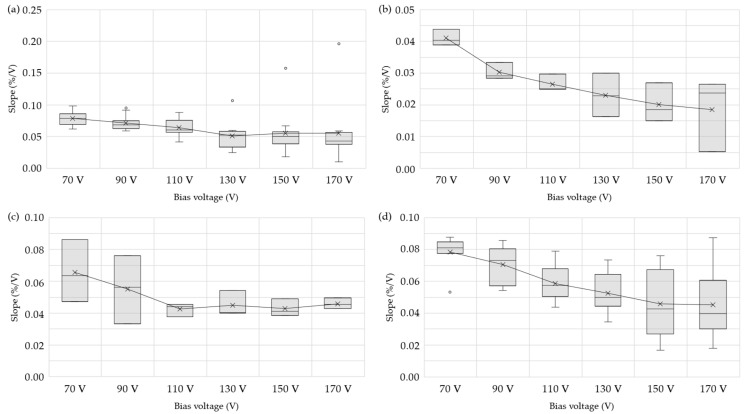
Slope of each detector model with different bias voltages: (**a**) micro-ionization chambers CRGR10/C5B/UG2, (**b**) micro-fission chambers CFUR44/C5B-U8/UG2, (**c**) micro-fission chambers CFUR43/C5B-U5/UG2, and (**d**) micro-fission chambers CFUR43/C5B-U8/UG2.

The average response and standard deviation for each detector model at various bias voltage are presented in Figure 5. The plateau region was obtained seeking the minimum slope in the response of the high-voltage sweep. It was calculated as the linear estimation of every four current values normalized to their average, linking the result with the central voltage of each interval. The statistical study of this data is displayed in a box-and-whisker plot (Figure 6). The box represents the interquartile range, and the whiskers extend to the most extreme data points within 1.5 times this range. Data beyond this threshold are considered outliers and shown as isolated round points in Figure 6. The outliers in Figure 6a obtained from IC number 206. Although it passed the acceptance test, this behavior may be attributed to manufacturing tolerances. The median is represented as a line inside the box, and the average is depicted as an “x”, connected by a solid line to see the trend of the slope across the voltage range. It can be inferred that the plateau region in these chambers can be found from 130–150 V onwards. Although this region appears to be optimal, nearly all voltages in the range between 70 V and 170 V exhibit an almost flat slope, which may be acceptable depending on the required level of precision. This confirms the indication of the manufacturer to polarize the devices at 150 V and lays the groundwork for conducting subsequent tests.

#### 3.1.3. Sensitivity of the Response to an X-Ray Tube

The sensitivity of the detectors was calculated as the slope of the linear regression of the response to the photons from an X-ray beam. The X-ray tube was kept at a voltage of 150 kV, varying the intensity between 0.5 mA and 22.5 mA corresponding to a dose rate from 0.05 to 2.28 Gy/min. DUTs were biased at 150 V, as it was consistent with the previous study.

**Figure 7:**
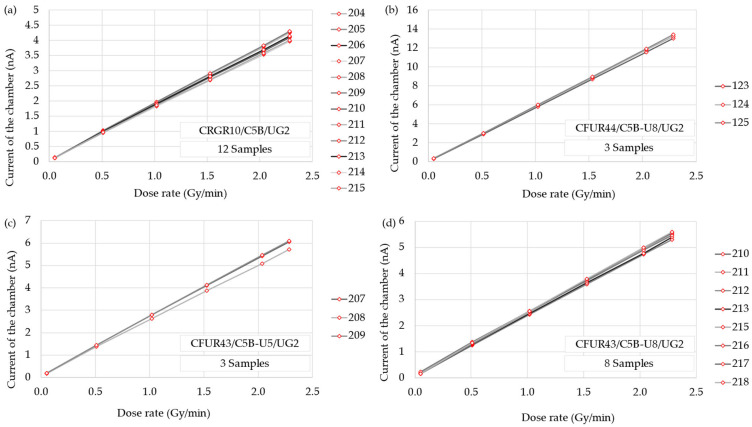
Response of every sample of each detector model to different dose rates of the X-ray tube: (**a**) CRGR10/C5B/UG2, (**b**) CFUR44/C5B-U8/UG2, (**c**) CFUR43/C5B-U5/UG2, and (**d**) CFUR43/C5B-U8/UG2.

Figure 7 shows the response of every sample of each detector model as a function of the applied dose rate. The response was linear to the dose rate, as the R^2^ coefficients were close to unity for all models. In Table 3, the sensitivity of the detectors was calculated as the average of the slope of the linear fit for each detector, and the uncertainty with a coverage factor k = 1.

Table 4 shows the uncertainties affecting the result of the experiment.

### 3.2. Monte Carlo Simulations

The numerical results obtained with the available chamber geometries are summarized in Table 5, where the energies absorbed in the active volume of the chambers are given for the different situations analyzed.

The results obtained in the simulations carried out with the simplified chamber geometries and the 150 kVp X-ray beam are shown in Table 6, which are very similar to those shown in Table 5 for the complete geometries.

## 4. Discussion

Based on the obtained data, we can draw several conclusions. First, the proposed setup and connection for signal acquisition to the chamber are suitable. The leakage current measured is less than 20 pA in the worst case, which is in accordance with the manufacturer’s guaranteed performance of 150 pA. The recommended operating voltage of 150 V, as specified by the manufacturer, was also validated. The plateau region was observed to start around 130–150 V. This result aligns with previous studies conducted with similar devices, in which they were biased at this voltage. Another point of interest is determining the breakdown voltage, at which the device ceases to operate within the plateau region. This information could provide additional insights.

The linearity of the response of each model was studied using an X-ray tube. The coefficients of determination of the linear fit of the response, R^2^, were close to unity for all models, thus confirming the linear behavior of the detectors for the X-ray tube intensity, which is related to the radiation dose rate emitted. Consequently, a sensitivity value for the photons generated by this tube was determined, obtaining 29.8±0.3 pA/(Gy/h) with the model CRGR10/C5B/UG2 chamber; 96.0±0.8 pA/(Gy/h) with CFUR44/C5B-U8/UG2; 43.0±0.9 pA/(Gy/h) with CFUR43/C5B-U5/UG2; and 39.2±0.3 pA/(Gy/h) with CFUR43/C5B-U8/UG2, with the devices polarized at 150 V. It can also be observed that the sensitivity increases in the model where the filling gas pressure is higher, as expected, resulting in a sensitivity increase by a factor of approximately 2.5–2.3. The test reproducibility was robust, as evidenced by the deviations presented in Table 7, computed relative to the mean slope of the linear fits for each detector, with a maximum deviation below 4.5%. Notably, the fission chambers with U-238 exhibited reduced data dispersion. This intra-model variability was also present in the acceptance test documentation of each chamber provided by the manufacturer.

On the other hand, although no study of long-term behavior was carried out in this work, according to a previous work [20] with similar fission microchambers, no significant sensitivity degradation may be expected.

Table 8 shows a comparative analysis of the response of several IC and FC detectors. Last four models correspond with those under study in this work, and the rest of the specifications are provided by the manufacturers. We can observe an excellent performance in terms of sensitivity with similar or even lower detection volume.

Monte Carlo simulations provided more insights into these experimental studies. In the case of the X-ray beam, the energy absorbed increases in the fission chamber with respect to the ionization one. This is in agreement with the measurements’ results, assuming that the energy absorbed is highly correlated to the produced photocurrent (device sensitivity). In the simulations, there is a factor ~1.5 between fission and ionization chambers, in line with that obtained in the experiment, 1.33. Additionally, increasing the gas pressure to 15 bar (chamber model CFUR44/C5B-U8/UG2) results in a 2.6 factor increase in the absorbed energy, closer to the sensitivity increment of 2.4 shown in Table 3. If we assume that the gas in the chambers behaves as an ideal gas, the ideal gas law establishes a proportional relationship between pressure and gas density: For constant volume and temperature, a higher pressure results in a higher gas density. Additionally, it is well known that an increase in gas density leads to a higher number of photon interactions within the gas, thereby enhancing the signal of the ionization chamber. In summary, an increase in gas pressure results in a higher signal from the studied device. This, at least partially, explains the differences observed between the signals of the CFUR44/C5B-U8/UG2 and CFUR43/C5B-U8/UG2 fission chambers. In case of the ^60^Co beam, the increase disappears and a slight reduction in the absorbed energy is even observed for the fission chamber with respect to the ionization one. This was already observed in the experimental results shown in [30].

Regarding the comparison between the simulations of the available geometry (Table 5) and the simplified one (Table 6), since the detailed geometry (materials and dimensions) of the various chambers was not available, simulations using the full precise geometry can only be considered an approximation. While the use of simplified geometries may seem unnecessary, in this context, it helps to determine whether the differences observed in the Monte Carlo simulations with the precise geometries are due to variations in the active volume elements (such as size and U-layer) or other factors. This simplified analysis has allowed us to confirm that these components are responsible for the discrepancies in signal between the ionization and fission chambers. Therefore, it can be concluded that, in the absence of the U layer, the increase in the absorbed energy disappears. Therefore, it is precisely the U layer of the fission chamber that is responsible for the enhancement in the absorbed energy and the physical mechanism provoking it is the photoelectric photon absorption, a process whose cross-section grows extremely quickly with the atomic number of the material, Z, being proportional to Z^4^ [8].

## 5. Conclusions

Experimental characterization and numerical simulation have been performed on different types of ionization and fission chambers with the same geometry but different anode and filling gas. The average leakage current obtained with the DUTs studied was 14 ± 3 pA, with a bias voltage of 150 V and X-ray tube conditions of 150 kV and 9 mA, which is much lower than the 150 pA accepted by the manufacturer, and allows for validating the setup and connections for subsequent tests.

The plateau region has been determined by studying the response of the devices to different bias voltages under consistent X-ray tube conditions. Acceptable values were observed across the voltage range from 70 to 170 V, with a notable improvement in response observed from 130 to 150 V onwards. It establishes the operating conditions under which the detectors must function to ensure reliable measurements, being consistent with the specifications provided by the manufacturer. The voltage at which the behavior of the devices transitions into the proportional region should be studied in the following tests.

The response of the DUTs biased at 150 V was linear to the current applied to the X-ray tube, obtaining an average sensitivity of 29.8±0.3 pA/(Gy/h) with the model CRGR10/C5B/UG2 chamber; 96.0±0.9 pA/(Gy/h) with CFUR44/C5B-U8/UG2; 43.0±0.8 pA_chamber_/mA_tube_ with CFUR43/C5B-U5/UG2; and 39.2±0.3 pA/(Gy/h) with CFUR43/C5B-U8/UG2. These differences in the chamber’s sensitivities have been explained with Monte Carlo simulations by the enhanced photoelectric photon absorption of the fission chambers under the experimental X-ray and with the increase in the filling gas pressure. This effect disappears with higher energy photons from ^60^Co, where photon energy loss by photoelectric effect is less important. Moreover, intra-model sensitivity variation has been calculated, showing a robust test of reproducibility, mainly in the fission chambers with U-238.

Future work will focus on studying the difference in response of ionization and fission chambers to neutron radiation, as fission chambers are specifically designed to detect them. Additionally, further research will explore their response to higher-energy photons and assess their degradation and stability under long and more demanding environmental conditions.

## Figures and Tables

**Table 1:** Detectors and main characteristics.

Model	Type	L_d_ (mm)	V_d_ (cm^3^)	N ^(a)^	Numbering	Fissile Material	Gas	Gas Pressure (Bar)
CRGR10/C5B/UG2	IC	14	0.025	12	204 to 215	-	Argon	5
CFUR44/C5B-U8/UG2	FC	10	0.018	3	123 to 125	U-238	Argon + 4% Nitrogen	15
CFUR43/C5B-U8/UG2				8	210 to 213 and 215 to 218	U-238	Argon	5
CFUR43/C5B-U5/UG2				3	207 to 209	U-235	Argon	5

^(a)^ Number of samples.

**Table 2:** Voltage sweeps applied.

Type of Sweep *	Range (V)	Step (V)	Measurements Taken	Expected Region
Low-voltage sweep	0–10	2	6	Recombination region
High-voltage sweep	0–200	20	21	Plateau region

(*) In both sweeps, the voltage pulses lasted 5 s. The signal was integrated for 1 s after allowing 3 s for the stabilization of the signal. A final rest period of 1 s was included before the next pulse.

**Table 3:** Sensitivity of the chambers at 150 V.

Model	Sensitivity (pA/(Gy/h))
CRGR10/C5B/UG2	29.8 ± 0.3
CFUR44/C5B-U8/UG2	96.0 ± 0.8
CFUR43/C5B-U8/UG2	39.2 ± 0.3
CFUR43/C5B-U5/UG2	43.0 ± 0.9

**Table 4:** Uncertainties of the experimental measurements for each detector model with a coverage factor of k = 1.

Type A ^1^	
CRGR10_C5B_UG2	0.6%
CFUR44_C5B_U8_UG2	0.11%
CFUR43_C5B_U5_UG2	0.5%
CFUR43_C5B_U8_UG2	0.5%
Type B ^2^	0.2%

^1^ Uncertainty of the sensitivity estimation, one for each detector model. ^2^ Uncertainty of the electrometer B2985B for operation at 20 nA range, common to all devices.

**Table 5:** Results obtained for the complete chamber geometries. The energy, per initial photon, absorbed in the active volume of the chambers is given. The numbers between parentheses are the uncertainties with a coverage factor k = 3.

	Absorbed Energy per Emitted Photon (eV)	
Chamber	150 kVp	^60^Co
Ionization (Ar 5 bar)	0.1214 (11)	0.1896 (14)
Fission (Ar 5 bar)	0.1990 (15)	0.1374 (10)
Fission (Ar + 4% N_2_ 15 bar)	0.523 (3)	0.410 (2)

**Table 6:** Results obtained for the simplified chamber geometries and the 150 kVp X-ray beam. The energy, per initial photon, absorbed in the active volume of the chambers is given. The numbers between parentheses are the uncertainties with coverage factor k = 3.

	Absorbed Energy per Emitted Photon (eV)
Chamber (Ar 5 Bar)	
Ionization	0.1190 (11)
Fission with U	0.1934 (15)
Fission without U	0.0835 (9)

**Table 7:** Deviation of the measurements with respect to the average of the slopes of the linear fit of the datasets for each chamber.

Detector Model	Average Relative Deviation (%)	Maximum Relative Deviation (%)	Average Absolute Deviation (pA/(Gy/h))	Maximum Absolute Deviation (pA/(Gy/h))
CRGR10_C5B_UG2	2.4	4.4	0.7	1.3
CFUR44_C5B_U8_UG2	1.0	1.5	1.0	1.5
CFUR43_C5B_U5_UG2	2.8	4.2	1.2	1.8
CFUR43_C5B_U8_UG2	1.7	2.8	0.7	1.1

**Table 8:** Main characteristics of commercial detectors. Last four correspond to models under study in this work.

Detector Model	Type of Detector	Manufacturer	Sensitive Volume (cm^3^)	Bias Voltage (V)	Response
Farmer Ionization Chamber 30013 waterproof	IC	PTW, Freiburg, Germany	0.6	±400 V	20 nC/Gy
PinPoint 3D IC (31022)	IC	PTW, Freiburg, Germany	0.016	±300 V	400 pC/Gy
FC65	IC	IBA Dosimetry, Schwarzenbruck, Germany	0.65	±300 V	21 nC/Gy
CC04	IC	IBA Dosimetry, Schwarzenbruck, Germany	0.04	±300 V	1 nC/Gy
FC165	FC	Centronic, Croydon, United Kingdom	-	±400 V	0.12 cps/nv *
CRGR10/C5B/UG2	IC	Photonis, Mérignac, France	0.025	+150 V	107 nC/Gy
CFUR44/C5B-U8/UG2	FC	Photonis, Mérignac, France	0.018	+150 V	346 nC/Gy
CFUR43/C5B-U8/UG2	FC	Photonis, Mérignac, France	0.018	+150 V	141 nC/Gy
CFUR43/C5B-U5/UG2	FC	Photonis, Mérignac, France	0.018	+150 V	156 nC/Gy

* Counts per second per neutron flux.

## Data Availability

The data presented in this study are available on request from the corresponding author.
